# A Patient With Schizophrenia in Remission Relapses Following COVID-19: A Case Report

**DOI:** 10.7759/cureus.29845

**Published:** 2022-10-02

**Authors:** Saeed S Shaaban, Rooble Bhullar, Ibrahim Mohammad, Aqeel Hashmi

**Affiliations:** 1 Department of Medicine, King Abdulaziz University, Jeddah, SAU; 2 Department of Psychiatry and Behavioral Sciences, Dayanand Medical College and Hospital, Ludhiana, IND; 3 Department of Psychiatry, Hawler Psychiatric Hospital, Erbil, IRQ; 4 Department of Behavioral Health, Westpark Springs, Houston, USA

**Keywords:** covid-19, treatment-resistant schizophrenia, atypical antipsychotic, schizophrenia relapse, psychosis, clozapine

## Abstract

As managing COVID-19 complications has become more prevalent in psychiatry, its effects can range from provoking new illnesses in previously healthy individuals to inducing relapses in patients in remission. However, an aspect of COVID-19’s influence that is not well documented is its effect on medication responsiveness. In this case, we present a 28-year-old male diagnosed with treatment-resistant schizophrenia for eight years. While in remission on a maintenance dose of clozapine, he was admitted to the hospital with signs of severe psychosis after testing positive for COVID-19. On admission, he did not have any other major stressors and no prior comorbidities that could have induced the relapse. Despite being on a higher dose of clozapine for four weeks while hospitalized, the patient’s psychosis did not improve. This raises the question if his infection had altered his response to medication that previously brought on remission.

## Introduction

Schizophrenia is a chronic recurrent psychotic disorder that is commonly associated with psychosocial impairment. Its presentation revolves around the presence of positive and negative symptoms, in combination with social and occupational impairment for at least six months [1]. The course of schizophrenia is unpredictable - roughly half of the patients reach a degree of functional remission [2]. Nevertheless, many patients are affected by recurrent relapses that might lead them to develop worsening symptoms and impaired psychosocial functioning, thereby increasing the burden of illness on them and their families [3].

As COVID-19 was first reported in 2019 [4], it has become a major concern for mental health providers. Recent studies have shown an increased association between psychiatric disorders and COVID-19 [5,6]. Furthermore, studies have revealed that patients with psychiatric disorders are at higher risk of being infected by COVID-19 [7,8], and clinicians have noticed the emergence or relapse of many psychiatric illnesses [8,9].

In this case report, we explore the case of a 28-year-old male patient diagnosed with schizophrenia who relapsed following COVID-19 infection and failed to respond to clozapine, which had induced remission in the past.

## Case presentation

The patient is a 28-year-old, single, South Asian male, with a history of treatment-resistant schizophrenia for eight years. He was previously in remission for two years before this relapse and had been gainfully employed full-time. During that period, he was stable on clozapine (100 mg), which had been reduced from 300 mg to reach a maintenance dose that was both efficacious and tolerable. His past psychiatric history shows multiple episodic relapses and more than 10 hospitalizations with minimal or no response to multiple antipsychotics, including risperidone, aripiprazole, paliperidone, ziprasidone, and olanzapine, despite the fact he was adherent to these medications. He did not have any disruptions in the continuity of treatment throughout the pandemic and was compliant with medication and blood tests due to support from his family. His past medical, surgical, and family histories are unremarkable.

The patient was initially taken to an inpatient facility for the episode of psychosis and aggression after developing high-grade fever (103 °F) with chills and loss of taste and testing positive for COVID-19 at home. He was placed on quetiapine and divalproex sodium for three days due to the absence of his treatment history but then changed to clozapine (200 mg) once daily and divalproex sodium (500 mg) twice per day after they were informed of his prior response to clozapine. He was discharged after 10 days of hospitalization and referred to our hospital’s day program.

However, on presentation to the day program, his speech was loud, rapid, and disorganized. His thought process was tangential, and he was still affected by poor judgment and insight. It became apparent that he was still being affected by persistent psychosis and aggressive behavior and required further inpatient treatment. The dose of clozapine was increased to 400 mg daily while divalproex sodium was discontinued. After two weeks of hospitalization without any improvement, oral haloperidol (5 mg) three times a day and a long-acting haloperidol decanoate injection (100 mg) were added to help with medication adherence due to severe paranoia. There has been a minimal response to this treatment in terms of psychosis. He still appeared preoccupied with religious delusions and paranoid explanations regarding life events. Additionally, he was extremely uncooperative and showed a hostile attitude toward the treating psychiatrist.

## Discussion

When managing psychiatric patients in the age of COVID-19, physicians should take extra precautions when dealing with patients with schizophrenia. Increased prevalence of COVID-19 infections and severe presentations have been well documented in the literature in patients with schizophrenia [10,11]. Even on maintenance antipsychotics, patients can still relapse after a period of stability [12]. Those with a more chronic course history are at greater risk despite ensured treatment adherence [13].

During our patient’s remission period, the dose of clozapine had been slowly tapered from 300 to 100 mg daily due to sialorrhea and complaints of sedation. This could have contributed to the current psychosis. However, he had been stable for more than six months on the lower dose, which raises the question if COVID-19 might have caused the nonresponse to clozapine. Multiple studies have shown how COVID-19 infection alters brain biology, altering both systemic and intracranial immune responses and developing an autoimmune response driven by the virus, thereby increasing the risk of psychiatric illness [14,15]. However, insufficient data is available about its impact on antipsychotic response.

Another important aspect of managing this case was the failure to respond to the medication that caused remission in the past. This places the patient at an increased risk of relapse and hospitalization. Coupled with each relapse, the patient's response rate gradually declines, putting them at a higher degree of disability and ultimately poorer outcome [16,17].

With clozapine being an integral part of managing treatment-resistant schizophrenia [18], it is important to shed light on this topic. Subsequently, extra precaution and adherence to preventive measures (social isolation, wearing a mask, and handwashing) are highly recommended to prevent infection. Patients and their families should be educated about the risk of relapse if they tested positive for COVID-19 and to seek psychiatric help even for mild symptoms or, as a precaution, set up an earlier visit with a mental health professional [19].

## Conclusions

With the expansive knowledge about the effects of COVID-19, more research is needed to study its effect on both relapse and response to antipsychotics in schizophrenic patients. This would help physicians plan and develop a strategy for managing patients and help give more insight into the effect of COVID-19 on the brain.
